# Slow-Onset Epidural Anesthesia for Cesarean Delivery in Severe Multivalvular Heart Disease and Atrial Fibrillation: The Role of Preoperative Optimization and Hemodynamic-Guided Management

**DOI:** 10.7759/cureus.99566

**Published:** 2025-12-18

**Authors:** Maria Massá Castro, João Gonzalez, Filipa Lança

**Affiliations:** 1 Anesthesiology, Unidade Local de Saúde de Santa Maria, Lisbon, PRT

**Keywords:** atrial fibrillation, cesarean section, epidural anesthesia, hemorrhagic risk, high-risk pregnancy, maternal cardiac disease, mitral stenosis, multiparity, multivalvular disease, rheumatic heart disease

## Abstract

Pregnancy places substantial stress on the cardiovascular system, and this effect is amplified in females with rheumatic valvular disease. We describe the anesthetic management of a 37-year-old multiparous patient with severe multivalvular disease (severe mitral stenosis, severe aortic insufficiency, and severe tricuspid regurgitation with stenosis) and atrial fibrillation, presenting with signs of congestive heart failure, who was submitted to an elective cesarean section at 37 weeks of gestation. Following admission, she received targeted medical optimization in a cardiac intensive care setting, including rate control and diuretic therapy, with subsequent clinical improvement. Anticoagulation with low-molecular-weight heparin was suspended 24 hours before surgery. Considering her fixed cardiac output and the potential harm of abrupt hemodynamic shifts during surgery, a slow-onset epidural anesthesia under invasive monitoring was performed. Incremental boluses of 0.75% ropivacaine, combined with early vasopressor support, allowed for an adequate sensory block, maintaining hemodynamic stability throughout surgery. Intraoperative tachyarrhythmia was managed through cautious fluid administration and pharmacological treatment. Oxytocin was carefully and slowly administered to prevent sudden reductions in systemic vascular resistance. Since resumption of therapeutic anticoagulation was planned due to high thromboembolic risk, the epidural catheter was removed at the end of the surgery. A multimodal analgesic strategy was adopted, including a single epidural morphine bolus and bilateral transversus abdominis plane blocks. Both maternal and neonatal outcomes were favorable. This case highlights the importance of meticulous preoperative optimization and planning, the safety of graded neuraxial anesthesia, and the need for multidisciplinary discussion when managing parturients with advanced multivalvular heart disease.

## Introduction

Cardiovascular disease represents a leading cause of fetal and maternal morbimortality during pregnancy, since the physiological adaptations of gestation are aggravated by significant valvular or rhythm abnormalities. The 2025 European Society of Cardiology (ESC) guidelines emphasize that the management of cardiovascular disease in pregnancy requires a multidisciplinary approach, integrating individualized risk stratification, pre-delivery optimization, and continuous hemodynamic assessment throughout the peripartum period [1].

Rheumatic heart disease (RHD) remains a major cause of cardiovascular morbidity during pregnancy worldwide, particularly in low and middle-income countries, where limited access to early diagnosis and long-term follow-up contributes to advanced valvular involvement at presentation. Globally, it affects an estimated 30-40 million individuals. Multivalvular involvement is common and increases maternal risk during pregnancy. In patients with previously compensated cardiac function, the physiological changes of pregnancy, such as increased circulating blood volume, elevated cardiac output, structural cardiac remodeling, and decreased systemic vascular resistance, can precipitate acute decompensation and heart failure, since valvular stenosis can limit the ability of the heart to accommodate larger blood volumes. These hemodynamic shifts pose unique challenges for the anesthesiologist, particularly during cesarean delivery, where rapid variations in preload (the venous return to the heart) and afterload (systemic resistance against which the heart ejects blood) can severely compromise cardiac function [2].

Recent literature has highlighted the importance of comprehensive perioperative management of these cases, including the use of invasive hemodynamic monitoring, vasoactive drug titration, and, in extreme cases, standby extracorporeal membrane oxygenation (ECMO) support [3,4]. Both regional and general anesthesia have been safely used; therefore, the choice should take into consideration the type and severity of the condition, as well as the anticipated hemodynamic impact of each technique. A tailored, multidisciplinary strategy is crucial to minimize maternal and fetal risk. General anesthesia and single-shot spinal techniques can induce rapid hemodynamic changes. In contrast, the use of epidural blockade allows gradual sympathetic blockade, with tighter control of blood pressure and heart rate [3-5].

Despite several reports addressing anesthesia in isolated valvular lesions, evidence remains limited regarding optimal anesthetic strategies in patients with combined severe multivalvular disease and atrial fibrillation, particularly those presenting with decompensation late in pregnancy. We report the anesthetic management of an elective cesarean section in a female with severe multivalvular RHD and atrial fibrillation, with the aim of highlighting perioperative optimization, hemodynamic-guided anesthetic planning, and the role of slow-onset epidural anesthesia in this high-risk population.

## Case presentation

A 37-year-old, African, multiparous female (gravida 5 para 4, three previous vaginal deliveries and one cesarean section 11 years before the current presentation), was admitted to the obstetric emergency department at 35 weeks + six days of gestation, after being evacuated from Cape Verde with the diagnosis of severe mitral stenosis. She had a known history of RHD since 2022, but had abandoned follow-up and medication. During the 3rd trimester of her current pregnancy, she developed palpitations, fatigue, asthenia, and peripheral edema, which progressively worsened. A diagnosis of atrial fibrillation was established, with an echocardiogram revealing trivalvular disease, characterized by severe mitral stenosis, moderate aortic regurgitation, and tricuspid valve disease, accompanied by biatrial dilation. Anticoagulation with warfarin was initiated, and the patient was transferred to Portugal due to the severity of her valvular disease.

On admission to our hospital, she was hemodynamically stable but tachycardic (heart rate = ~100 bpm) and exhibited signs of congestive heart failure, namely, peripheral edema and pulmonary congestion. Echocardiography confirmed severe multivalvular rheumatic disease. The aortic valve had rheumatic thickening with mild stenosis (mean gradient = 15 mmHg) and severe regurgitation (effective regurgitant orifice area (EROA) = 0.39 cm²; pressure half-time (PHT) = 320 ms; diastolic flow reversal in the thoracic aorta). The mitral valve showed rheumatic morphology with moderate stenosis (mean gradient = 7 mmHg; estimated valve area = 1.8 cm²) and moderate regurgitation. The tricuspid valve had severe regurgitation (vena contracta = 8 mm; EROA = 0.94 cm²; regurgitant volume = 48 mL) with associated stenosis (mean gradient = 7 mmHg). Chambers showed severe biatrial dilation with preserved biventricular systolic function. The inferior vena cava (IVC) was dilated (27 mm) with reduced respiratory variation.

She was admitted to the cardiac intensive care unit (CICU) for cardiovascular optimization and monitoring, and an elective cesarean section was planned for 37 weeks of gestation. The patient was started on rhythm control therapy with digoxin 0.25 mg daily and diuretic therapy with furosemide (at first, 40 mg every eight hours, and then reduced to 20 mg every eight hours), and warfarin was switched to therapeutic low-molecular-weight heparin (LMWH) 1 mg/kg every 12 hours.

During the CICU stay, progressive clinical improvement was observed, with resolution of dyspnea and lower-limb edema. A pre-anesthetic assessment was conducted, and the case was discussed with the cardiologist specialized in pregnancy-related cardiovascular disease to determine optimal perioperative management and anesthetic strategy.

LMWH was discontinued 24 hours before surgery, and furosemide was withheld on the morning of the procedure. Upon arrival to the operating room, the patient was in atrial fibrillation with rapid ventricular response (heart rate = 110-120 bpm), normotensive, and eupneic with no signs of respiratory distress.

Before anesthetic induction, we inserted an arterial catheter for invasive pressure monitoring and blood gas analysis, along with a right internal jugular central venous line. An epidural catheter was placed at the lumbar level and tested with 3 mL of 2% lidocaine. Thereon, incremental boluses of 4 mL of 0.75% ropivacaine were given each time, supplemented by a single dose of 10 mcg of sufentanil. A cautious crystalloid co-load fluid administration was provided, and a low-dose norepinephrine perfusion was started preemptively. With this approach, we achieved a sensory block up to T6 within 40 minutes, without significant hemodynamic repercussion. Blood pressure remained within normal limits throughout induction and surgical preparation, with invasive arterial monitoring allowing continuous assessment and early intervention. The neonatal Apgar score was 9/10/10.

After delivery, uterotonic therapy was initiated with a carefully administered 10 IU oxytocin bolus, followed by a continuous infusion of 5 IU over one hour. A transient hypotensive episode occurred, promptly managed by titrating norepinephrine dosing.

Intraoperatively, rapid ventricular response was aggravated, with the patient reaching heart rates of 130-140 bpm, although hemodynamic stability was maintained. Firstly, a 20 mg esmolol bolus was given, providing only brief rate control, followed by the administration of 2 g of magnesium sulfate and 0.25 mg of digoxin. Arterial blood gas analysis revealed no electrolyte disturbances, but a hemoglobin level of 18.7 g/dL and hematocrit of 50% stood out. Even though urine output remained adequate, urine appeared concentrated. Intravenous fluid administration was cautiously increased to correct relative hypovolemia that could be contributing to the tachycardia. These measures effectively reduced the heart rate to 100-105 bpm, and the patient remained stable, allowing norepinephrine to be discontinued by the end of the surgery. Respiratory function was stable throughout, without the need for supplemental oxygen or ventilatory support.

For postoperative pain control, a multimodal approach was adopted. As resumption of therapeutic anticoagulation was planned due to high thromboembolic risk, the epidural catheter was removed at the end of the surgery. An epidural bolus of 1 mg morphine was administered before removing the catheter. Intravenous analgesia with acetaminophen (1 g), ketorolac (10 mg), and tramadol (100 mg) was complemented with bilateral transversus abdominis plane (TAP) blocks using 40 mL of 0.2% ropivacaine per side.

Postoperatively, the patient was transferred back to the CICU for close monitoring. Her clinical course was favorable, without post-surgical complications and maintained hemodynamic stability, having been discharged home five days later, referred to cardiothoracic surgery for planned aortic and mitral valve replacement with tricuspid valve repair.

## Discussion

Severe multivalvular rheumatic disease in pregnancy represents a hemodynamic challenge, given the profound alterations in cardiovascular physiology that occur in gestation, including increased circulating blood volume, augmented cardiac output, and decreased systemic vascular resistance, which may precipitate decompensation in patients with pre-existing valvular disease, especially when accompanied by atrial arrhythmias [1,6,7]. Our patient presented the combination of severe mitral stenosis, aortic regurgitation, severe tricuspid stenosis and insufficiency, as well as atrial fibrillation, thus posing a high risk of pulmonary congestion, reduced cardiac reserve, and perioperative hemodynamic instability. Notably, triple-valve rheumatic involvement in pregnancy is uncommon, and the coexistence of both tricuspid stenosis and regurgitation further complicates preload management and right-sided cardiovascular physiology.

On the other hand, advanced age, multiparity, and previous cesarean section are recognized risk factors for postpartum hemorrhage (PPH), primarily due to increased likelihood of uterine atony, thus augmenting obstetric risk and the complexity of the case [8,9].

In patients with severe cardiac disease, the occurrence of significant blood loss or rapid intravascular volume shifts may lead to heart failure decompensation or dysrhythmias. Moreover, standard obstetric management, namely, the administration of uterotonic drugs, can invoke abrupt vasodilation and negative inotropy, thus reducing systemic vascular resistance and preload, which might be poorly tolerated in the setting of fixed or limited cardiac output [10]. In this case, the transient hypotension observed following oxytocin administration reinforces the need for slow titration and vigilant hemodynamic monitoring when using uterotonics in parturients with advanced valvular disease.

Regarding anesthetic technique, the choice and execution must take into account the imperative of preventing cardiovascular decompensation. Neuraxial anesthesia, specifically epidural block, offers the advantage of providing adequate surgical anesthesia while allowing gradual sympathetic blockade, thereby minimizing abrupt hemodynamic changes compared with single-shot spinal anesthesia [11-13]. Several case series and reviews affirm the safety of epidural anesthesia in parturients with RHD when meticulously managed [11]. In our case, incremental ropivacaine boluses combined with sufentanil, alongside prophylactic low-dose norepinephrine infusion, allowed attainment of a sensory block to T6 within 40 minutes without significant hypotension - a key outcome given the patient’s fixed cardiac output state and limited ability to compensate for sudden reductions in preload or afterload [14,15]. Alternative anesthetic strategies were considered but deemed less suitable. General anesthesia may precipitate tachycardia during induction, reduce systemic vascular resistance with positive-pressure ventilation, and increase myocardial oxygen demand, all of which can worsen hemodynamic instability in fixed cardiac output states. Single-shot spinal anesthesia was also avoided because of the potential for abrupt sympathetic blockade and sudden decreases in preload and systemic vascular resistance, which could have precipitated severe hypotension and cardiovascular collapse. Given this patient’s severe multivalvular lesions, atrial fibrillation, and high risk of decompensation, a graded epidural technique was chosen to allow slow, controlled titration of sympathetic blockade.

Adequate fluid management is another critical aspect of care. Hypovolemia may impair cardiac output, whereas fluid overload increases the risk of pulmonary edema, particularly in the presence of biatrial dilation and tricuspid regurgitation. Invasive hemodynamic monitoring should be used, since it allows a more accurate management of preload, afterload, and heart rate [16,17]. The restrictive co-loading strategy used here, guided by invasive arterial monitoring and urine output, contributed to a more precise intravascular volume control. During surgery, mean arterial pressure was maintained within acceptable limits under low-dose norepinephrine support, while heart rate ranged between 110 and 140 beats per minute prior to rate control interventions. The episode of rapid ventricular response observed intraoperatively was clinically significant. In the setting of severe mitral stenosis, tachycardia markedly reduces diastolic filling time, leading to elevated left atrial pressures, worsening pulmonary congestion, and reduced forward cardiac output, thereby increasing the risk of hemodynamic decompensation. Rate control was therefore a central goal of management. The combined use of esmolol, magnesium sulfate, and digoxin, along with cautious fluid administration to correct relative hypovolemia suggested by hemoconcentration and concentrated urine, successfully reduced the heart rate to 100-105 beats per minute without precipitating volume overload [18].

Perioperative anticoagulation management required careful multidisciplinary coordination. Transition from warfarin to therapeutic LMWH and its suspension 24 hours before surgery was essential to balance thromboembolic and hemorrhagic risks. Postoperatively, early removal of the epidural catheter enabled timely resumption of anticoagulation, in accordance with current recommendations on neuraxial anesthesia in patients receiving antithrombotic therapy [19].

The multimodal analgesic regimen was adopted, which included a single epidural morphine bolus combined with bilateral TAP blocks, providing satisfactory postoperative pain control while minimizing systemic opioid requirements, aligning with the Enhanced Recovery After Cesarean (ERAC) protocols [20]. Adequate analgesia is particularly important in cardiac patients, as pain-related sympathetic activation may exacerbate tachycardia and hemodynamic instability.

The favorable outcome in this high-risk case highlights several important considerations for clinical practice: (1) meticulous preoperative optimization is essential; (2) graded neuraxial anesthesia with incremental dosing and vasopressor support can safely be employed in parturients with advanced valvular heart disease; (3) multiparity and hemorrhagic risk must be explicitly incorporated into anesthetic planning; (4) invasive monitoring and vigilant hemodynamic management are non-negotiable in this population; (5) the coexistence of severe multivalvular rheumatic involvement, including significant tricuspid regurgitation with stenosis and atrial fibrillation during pregnancy, is uncommon and underscores the need for individualized, multidisciplinary planning to optimize maternal and fetal outcomes.

This case report is limited by its single-patient design, which restricts generalizability. Management decisions were made based on the resources and context available, which may differ in other settings. As with all case reports, there is potential for reporting bias. Nevertheless, this case highlights important clinical insights into the perioperative management of high-risk obstetric patients with complex cardiac disease undergoing elective cesarean delivery.

## Conclusions

The anesthetic management of parturients with severe rheumatic multivalvular heart disease is challenging and requires an individualized and multidisciplinary approach. A slow-onset epidural block appears to be a safe strategy to minimize abrupt hemodynamic changes while providing adequate surgical anesthesia, and hemorrhagic risk must be carefully considered and prevented.

Although conclusions drawn from a single case report cannot be generalized, this case illustrates how graded neuraxial anesthesia, vigilant hemodynamic monitoring, and close collaboration between anesthesiology, cardiology, and obstetrics teams may contribute to safe perioperative management in high-risk obstetric patients with complex valvular disease.
